# Common genetic architecture underlying young children’s food fussiness and liking for vegetables and fruit^1^,^2^,^3^

**DOI:** 10.3945/ajcn.115.122945

**Published:** 2016-02-10

**Authors:** Alison Fildes, Cornelia HM van Jaarsveld, Lucy Cooke, Jane Wardle, Clare H Llewellyn

**Affiliations:** 4 Health Behavior Research Centre, Department of Epidemiology and Public Health, University College London, London, United Kingdom; and; 5 Department for Health Evidence and; 6 Department of Primary and Community Care, Radboud University Medical Center, Nijmegen, Netherlands

**Keywords:** child, eating, food, fussiness, genetic, heritability, infant, liking, preferences

## Abstract

**Background:** Food fussiness (FF) is common in early childhood and is often associated with the rejection of nutrient-dense foods such as vegetables and fruit. FF and liking for vegetables and fruit are likely all heritable phenotypes; the genetic influence underlying FF may explain the observed genetic influence on liking for vegetables and fruit. Twin analyses make it possible to get a broad-based estimate of the extent of the shared genetic influence that underlies these traits.

**Objective:** We quantified the extent of the shared genetic influence that underlies FF and liking for vegetables and fruit in early childhood with the use of a twin design.

**Design:** Data were from the Gemini cohort, which is a population-based sample of twins born in England and Wales in 2007. Parents of 3-y-old twins (*n* = 1330 pairs) completed questionnaire measures of their children’s food preferences (liking for vegetables and fruit) and the FF scale from the Children’s Eating Behavior Questionnaire. Multivariate quantitative genetic modeling was used to estimate common genetic influences that underlie FF and liking for vegetables and fruit.

**Results:** Genetic correlations were significant and moderate to large in size between FF and liking for both vegetables (−0.65) and fruit (−0.43), which indicated that a substantial proportion of the genes that influence FF also influence liking. Common genes that underlie FF and liking for vegetables and fruit largely explained the observed phenotypic correlations between them (68–70%).

**Conclusions:** FF and liking for fruit and vegetables in young children share a large proportion of common genetic factors. The genetic influence on FF may determine why fussy children typically reject fruit and vegetables.

See corresponding editorial on page 961.

## INTRODUCTION

It is common for young children to reject certain types of foods, especially those with certain textures or flavors [so-called food fussiness (FF)^7^]. Fussy eating behavior typically emerges in toddlerhood (1, 2) and can result in lower dietary variety and quality (3–5). The foods rejected most frequently by fussier children tend to be the nutrient-dense, healthier foods such as vegetables and fruit (6). Food neophobia, a distinct but related characteristic that refers specifically to the refusal of unfamiliar foods, is also associated with low vegetable and fruit acceptance (7–10). Because of the importance of vegetable and fruit consumption for health (11–13) and the low rates of vegetable consumption reported in children in developed countries (13–15), it would be valuable to gain a better understanding of the relation between fussy and neophobic behaviors and the rejection of vegetables and fruit specifically.

Studies that have explored the origins of food rejection have often focused on negative reactions to new foods (neophobia), but children may also start to refuse previously liked or accepted foods, thereby becoming increasingly selective in their eating (5), and these different types of food refusal are closely linked (7, 16). The 6-item FF scale from the Children’s Eating Behavior Questionnaire (CEBQ) is an established measure of children’s food rejection and measures both aspects of neophobia (e.g., “My child refuses new foods at first”) and more general fussy eating (e.g., “My child is difficult to please with meals”).

From twin studies, it has been well established that neophobia, when measured independently of other fussy behaviors, has a strong genetic basis (72–78%) in the early childhood period (17, 18). A substantial genetic influence has also been shown for young children’s preferences for vegetables and fruit with heritability estimates in the order of 37–54% (19, 20). One hypothesis is that part of the heritability of vegetable and fruit liking reflects the genetic influence on fussiness and neophobia and that a large proportion of the observed phenotypic association between vegetable and fruit liking and fussiness and neophobia is mediated by the genetic influence on fussiness and neophobia.

In the current study, we used pediatric twin data from the Gemini cohort to test this hypothesis. We used multivariate quantitative genetic analyses to quantify the extent to which genetic influences that underlie FF are the same as those that underlie vegetable and fruit liking and the extent to which common genetic influences explain the observed phenotypic associations between FF and vegetable and fruit liking.

## METHODS

### Sample

Data were from children participating in the Gemini twin study. Gemini is a birth cohort of twins born between March and December 2007 in England and Wales (21). All families with twins born in this period (*n* = 6754) were contacted by the Office for National Statistics and were invited to participate in the study. Of these, 2402 families returned a baseline questionnaire (36% response rate) when the twins were, on average (±SD), 8.2 ± 2.2 mo old (**Supplemental Figure 1**). This study used data from families who completed the measures of food liking and the CEBQ (22) when the children were aged 3.5 ± 0.3 y (*n* = 2686). Ethical approval was granted by the Joint University College London/University College London Hospitals Committee on the Ethics of Human Research.

### Measures

Zygosity was determined in same-sex pairs with the use of a validated questionnaire (23). In addition, DNA-based zygosity testing was conducted in a random sample of 81 Gemini pairs, which resulted in 100% correspondence between questionnaire-allocated and DNA-tested zygosity (24). In the current sample, zygosity was uncertain in 13 pairs because of inconclusive questionnaire results or missing data, and therefore, these pairs were excluded from the analyses. The sex, date of birth, weight at birth, and gestational age of the children were reported in the baseline questionnaire, and the exact age at the assessment of food liking and FF was calculated from the date of data collection.

The measure of food liking used in this study has been described previously (19). Briefly, parents reported their children’s liking for multiple individual foods on a 6-point scale with the following response options: likes a lot, likes, neither likes nor dislikes, dislikes, dislikes a lot, and never tried (the last category was recoded to missing). Responses were scored 2, 1, 0, −1, and −2; with zero indicating a neutral opinion, positive values indicating liking, and negative values indicating dislike. A total of 75 foods were grouped into 6 categories that were primarily derived from a principal components analysis. Foods were required to have been tried by ≥75% of children to be included in the principal components analysis. The food categories included in the current study were vegetables (19 foods; e.g., broccoli) and fruit (16 foods; e.g., bananas). Scale scores were calculated as the mean liking for the component food items. In order for a scale score to be calculated, participants were required to have completed greater than one-half of the food items within each scale.

Parents reported on their children’s fussiness with the use of the CEBQ FF scale, which was designed to assess neophobic, fussy, and picky eating behaviors in children (22). The 6 items (example item: “My child refuses new foods at first”) were scored on a 5-point scale labeled never, rarely, sometimes, often, or always. Mean scores were calculated for each child (range: 1–5) with higher scores indicating greater fussiness. Complete data were required on ≥4 items.

### Statistical analyses

Heritability was estimated with the use of intraclass correlations (ICCs) and maximum-likelihood structural equation modeling (MLSEM). Analyses were conducted on food liking and FF scores that had been residualized for age and sex effects with the use of a regression procedure. This method took into account the exact correlation for age (and sex within same-sex twin pairs) that could inflate the estimate of shared environmental effects (*C*) (25).

Twin studies make it possible to estimate the extent of genetic influence on a characteristic by comparing the degree of resemblance between monozygotic twin pairs (who share 100% of their genes) and dizygotic twin pairs (who share, on average, 50% of their segregating genes). ICCs provide an indication of the size of the genetic effect on a single characteristic, whereby the greater the resemblance between monozygotic and dizygotic twins, the larger the genetic influence on that trait. Cross-twin, cross-trait (CT/CT) correlations form the basis of multivariate heritability. They show how, within a twin pair, the score of twin 1 for trait A (e.g., FF) varies in relation to the score of twin 2 for trait B (e.g., liking for vegetables). Similar to simple ICCs, higher CT/CT correlations between monozygotic and dizygotic pairs indicate that shared genetic influences are driving the phenotypic association between the traits.

MLSEM was used to derive more-reliable estimates of genetic and environmental influences on the traits and the common influence between them and to provide 95% CI and goodness-of-fit statistics. MLSEM provides estimates for additive genetic effects (*A*), *C*, or unique environmental effects (*E*) by producing a large number of possible variable values and comparing them to the variance-covariance structures observed in the actual data in an iterative process. The estimates selected are those that produce variance-covariance structures that most closely resemble the actual data (26).

CT/CT ICCs were calculated for monozygotic and dizygotic pairs for the residualized vegetable- and fruit-liking scores paired with residualized FF scores. For each combination of food liking and FF, there were 2 CT/CT correlations as follows: *1*) FF in twin 1 was correlated with vegetable liking in twin 2, and *2*) vegetable liking in twin 1 was correlated with FF in twin 2. These correlations were compared with the phenotypic correlations calculated with the use of Pearson’s product-moment correlation coefficients to identify evidence of the underlying common genetic influence for both traits. Twin correlations were conducted with the use of SPSS version 21 for Windows software (SPSS Inc).

The MLSEM used a correlated factors model (26, 27). This model provides the following 2 pieces of information about shared genetic effects between measured phenotypes: *1*) pairwise etiologic correlations and *2*) bivariate heritability estimates. Etiologic correlations quantify the extent to which common genetic factors or common environments influence 2 phenotypes (e.g., vegetable liking and FF). Etiologic correlations can be interpreted in the same way as Pearson correlation coefficients such that a positive etiologic correlation indicates that the influences that cause an individual to score higher on one particular phenotype also tend to make them score higher on the other phenotype; in the same way, a negative correlation would indicate that the same influences that cause an individual to score higher on one phenotype tend to make them score lower on the other phenotype. Bivariate estimates quantify the extent to which common factors (*A*, *C*, or *E*) explain the observed phenotypic association (e.g., between vegetable liking and FF). We also tested submodels by systematically dropping components of variance (*A*, *C*, or *E*) and covariance, but all submodels led to a worsening of fit according to the likelihood ratio test and Aikaike’s information criterion. MLSEM was conducted with the use of Mx Maximum-Likelihood Structural Equation Modeling Software (version 32; Virginia Commonwealth University).

## RESULTS

Sample characteristics are provided in **Table 1**. The phenotypic correlations between FF and liking for vegetables and fruit are shown in **Table 2**. FF was significantly negatively correlated with liking for both vegetables (−0.61, *P* < 0.01) and fruit (−0.42, *P* < 0.01), such that fussier children tended to dislike vegetables and fruit. Sizes of the associations were moderate to large.

**TABLE 1:** Sample characteristics

Characteristic	Study sample (*n* = 2660 children)
Sex, *n* (%)	
M	1316 (49.5)
F	1344 (50.5)
Gestational age, wk	36.18 ± 2.51^1^
Weight at birth, kg	2.45 ± 0.54
Zygosity, *n* (%)	
Monozygotic	916 (34.4)
Dizygotic	1744 (65.6)
Food fussiness^2^	2.65 ± 0.85
Vegetable liking^3^	0.44 ± 0.61
Fruit liking^3^	1.00 ± 0.64

1 Mean ± SD (all such values).

2 Higher scores indicate greater fussiness (range: 1–5).

3 Higher scores indicate a higher liking (range: −2 to 2).

**TABLE 2:** Phenotypic and CT/CT ICCs for V and FF, and F and FF^1^

			CT/CT ICCs (95% CIs)^4^	
Scales	Phenotypic correlations^2^	Twin and scale^3^	Monozygotic	Dizygotic
Vegetable liking × FF	−0.61	Twin 1 V × twin 2 FF	−0.58 (−0.64, −0.52)	−0.29 (−0.35, −0.23)
		Twin 2 V × twin 1 FF	−0.58 (−0.64, −0.52)	−0.32 (−0.38, −0.26)
Fruit liking × FF	−0.42	Twin 1 F × twin 2 FF	−0.45 (−0.53, −0.38)	−0.20 (−0.26, −0.13)
		Twin 2 F × twin 1 FF	−0.42 (−0.50, −0.34)	−0.19 (−0.26, −0.13)

1 CT/CT, cross-twin, cross-trait; F, fruit liking; FF, food fussiness; ICC, intraclass correlation; V, vegetable liking.

2 Pearson’s product-moment correlation coefficients; *n* = 2523–2660. All correlations were significant at the 0.01 level (2 tailed).

3 Randomly allocated twin (1 or 2) and the scale used in the CT/CT correlation. CT/CT ICCs were calculated for monozygotic and dizygotic pairs for the residualized vegetable and fruit liking scores paired with residualized FF scores.

4 Monozygotics: *n* = 438–458 pairs; dizygotics: *n* = 855–872 pairs.

### Twin correlations

The pairwise CT/CT correlations between each of the food-liking scales and FF are also shown in Table 2. The CT/CT correlations between FF and both vegetable and fruit liking were significant (the 95% CIs did not cross 0) and moderate to large for monozygotic twins, whereas the dizygotic correlations, although still significantly different from zero, were considerably smaller. This pattern of high similarity indicated that shared genes were contributing to the observed phenotypic correlations between FF and vegetable liking and FF and fruit liking. In keeping with the phenotypic correlations, the CT/CT ICCs were negative, which indicated that, if one twin within a pair scored highly on FF, his or her co-twin tended to score lower on liking for fruit or vegetables.

### Multivariate MLSEM

**FIGURE 1:**
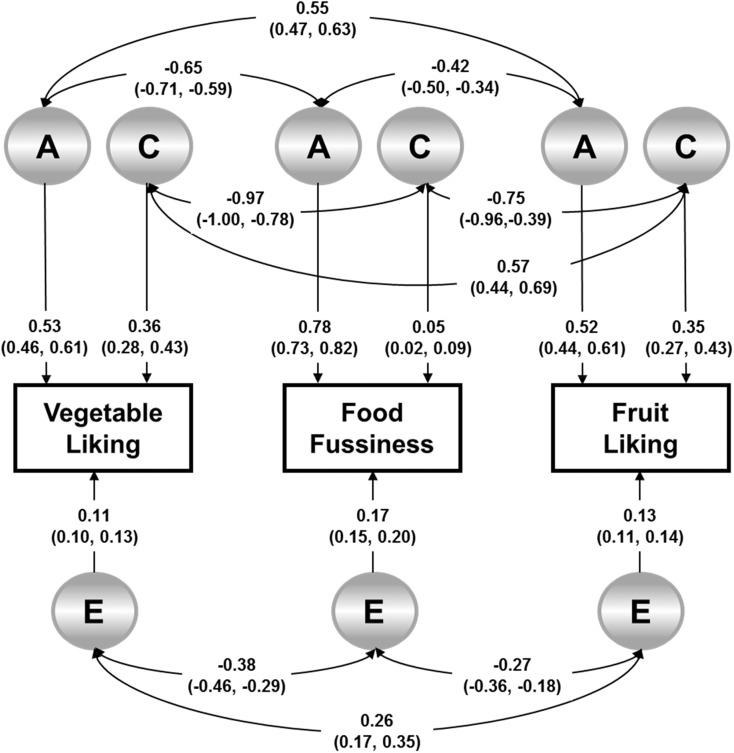
Full ACE-correlated factors model showing the genetic and environmental influences on children’s vegetable liking, fruit liking, and food fussiness. The path diagram shows the genetic and environmental influences on fruit and vegetable liking and food fussiness for one child with the use of a correlated factors model. Data were analyzed with the use of multivariate maximum-likelihood structural equation modeling. Each rectangular box represents the measured phenotype (food liking or food fussiness). Circles indicate latent influences on the measured phenotype, which included A, C, and E. Straight single-headed arrows show causal paths, and squared path coefficients on each causal path indicate the total variance explained in each trait by A, C, and E. The curved double-headed arrows show the genetic, shared environment and unique environment correlations between the traits. *n* = 2660 children. A, additive genetic effects; C, shared environmental effects; E, unique environmental effects and error.

**Figure 1** shows the univariate estimates for *A*, *C*, and *E* (single-headed straight arrows) derived for the 3 traits from the multivariate model as well as the etiologic correlations between them (double-headed curved arrows). The univariate estimates for FF established that it was highly heritable (*A*: 0.78; 95% CI: 0.73, 0.82) with the majority of the remaining variance being explained by unique environment effects (*E*: 0.17; 95% CI: 0.15, 0.20). The shared environment had a very small effect on FF (*C*: 0.05; 95% CI: 0.02, 0.09). Univariate estimates were virtually the same for vegetable liking and fruit liking. Heritability was moderate for both vegetable liking (*A*: 0.53; 95% CI: 0.46, 0.61) and fruit liking (*A*: 0.52; 95% CI: 0.44, 0.61) in keeping with estimates reported previously (19). Estimates of shared environmental influences were also moderate for both vegetable liking (*C*: 0.36; 95% CI: 0.28, 0.43) and fruit liking (*C*: 0.35; 95% CI: 0.27, 0.43).

The phenotypic correlations derived from the structural equation modeling, the pairwise etiologic correlations, and the bivariate estimates (the proportion of the phenotypic correlations explained by common genetic or environmental influences) are shown in **Table 3**. The genetic correlations between liking for vegetables and FF (−0.65) and liking for fruit and FF (−0.42) were significant and moderate to large, which indicated that many of the genetic factors that underlie FF also influence liking for both vegetables and fruit. The negative genetic correlation for vegetable liking and FF was significantly stronger than the association for fruit liking and FF, which showed that a particularly high proportion of the genetic influences that drive increased FF were also behind decreased liking for vegetables. This result supports the hypothesis that some of the genetic influence on liking for vegetables and fruit reflects a genetic influence on FF.

**TABLE 3:** Variable estimates for covariance and *A*, *C*, and *E* that underlie children’s vegetable liking, fruit liking, and FF^1^

		Variance components for bivariate *A*, *C*, and *E*, phenotypic correlation, % (bivariate estimates)^3^			Etiologic correlation (95% CI)^4^		
Food preference and FF scales	Phenotypic correlation (95% CI)^2^	*A*	*C*	*E*	*r*_g_	*r*_c_	*r*_e_
Vegetable liking × FF	−0.60 (−0.66, −0.55)	70 (0.42)	21 (0.13)	9 (0.05)	−0.65 (−0.71, −0.59)	−0.97 (−1.00, −0.78)	−0.38 (−0.46, −0.29)
Fruit liking × FF	−0.40 (−0.46, −0.36)	66 (0.26)	24 (0.10)	10 (0.04)	−0.42 (−0.50, −0.34)	−0.75 (−0.96, −0.39)	−0.27 (−0.36, −0.18)

1 *n* = 2660 children. *A*, additive genetic effects, *C*, shared environment effects; *E*, unique environment effects; FF, food fussiness; *r*_c,_ shared environmental correlation; *r*_e_, unique environmental correlation; *r*_g_, genetic correlation.

2 Phenotypic correlations were derived from structural equation modeling.

3 Proportions of variance in the phenotypic correlation that are explained by common *A*, *C*, and *E* derived from structural equation modeling and converted to percentages for ease of interpretation. The sum of the bivariate components (shown in parentheses) equals the phenotypic correlation. All bivariate estimates were significant.

4 A genetic, shared environmental or unique environmental correlation was significant if the 95% CI did not include zero; all correlations in the model were significant.

The bivariate heritability estimates (*A*) indicated significant genetic contributions to the phenotypic associations between liking for vegetables and FF and between liking for fruit and FF. The bivariate heritability estimates were similar for vegetable liking and FF (70%) and for fruit liking and FF (66%), which suggested that the majority of the phenotypic correlation between each of these pairs of traits could be ascribed to common genetic factors, thereby indicating that the majority of the observed phenotypic associations between FF and liking for both vegetables and fruit were genetically mediated.

## DISCUSSION

The results from this study support the hypothesis that a significant proportion of the genetic influence on liking for vegetables and fruit reflects a genetic influence on FF and that the majority of the observed phenotypic associations between FF and liking for vegetables and fruit are genetically mediated. These findings show that FF and liking for vegetables and fruit are heritable traits in young children and, also, that common genes are driving the association between fussy eating and decreased preferences for these nutritious foods. FF was shown to be highly heritable (78%) with only a moderate influence of the environment on this characteristic in young children. These results are comparable to findings from twin studies that investigated genetic and environmental influences on food neophobia, which have previously been estimated as 72% heritable in 4–7-y-olds and 78% heritable in 8–11-y-olds (17, 18).

The strong phenotypic associations observed between higher FF and lower liking for vegetables and fruit also support previous findings (3–5, 9). These associations were largely driven by genetic influences that were shared with FF (70% for vegetables and 66% for fruit). The phenotypic relation between liking for vegetables and FF was particularly strong, and shared genes appeared to be the largest contributor to this association. These results suggest fussy children display lower liking for vegetables primarily because both of these traits are driven by the same underlying genetic factors.

The findings of this study raise the following question: What are the common genetic factors driving the associations between these traits? Note that the food groups most commonly rejected by fussy eaters (i.e., vegetables, fruit, and, to a lesser extent, protein) (10) are also those for which liking is most heritable (19, 20). Research on the topic of genetically determined variation in human taste sensitivity has focused on sensitivity to the compounds phenylthiocarbamide and 6-*n*-propylthiouracil (PROP) and the associated taste 2 receptor member 38 (*TAS2R38*) gene. PROP sensitivity has been used as a marker for general taste acuity, and the most frequently applied test of human taste function has involved asking people to rate the intensity of this compound (28). The use of PROP taster status as a marker of general taste sensitivity or ability has received criticism (28, 29), and growing evidence has pointed to a complex etiology of taste perception or preference (30–32).

Traits such as food preferences and FF are likely to be highly polygenic with many genes each contributing a small amount to the genetic variation in these phenotypes. This contribution makes it difficult to identify the specific genes responsible although there has been progress in the detection of genetic influences on other complex polygenic traits such as obesity (33) and aspects of appetite, including satiety (34). To date, 97 genetic variants have been shown to contribute to the variation in body mass (35). The large genetic correlations observed between food liking and FF in this study suggest that, if the genes that contribute to the variation in fussiness were to be identified, they would also likely influence liking for vegetables and fruit. However, the etiologic correlations were not complete, which indicated that there was also some genetic heterogeneity in the 3 traits. Therefore, the wider search for genes that underlie food preferences would benefit from the measuring of the many dimensions that characterize taste sensitivity, oral sensitivity, and food rejection to obtain a complete picture.

Common shared environmental factors were also shown to contribute to the association between FF and vegetable liking (21%) and FF and fruit liking (24%). Although the shared environment only explained a small proportion of the variance in FF (5%), almost all of the shared environmental influences that contributed to FF also contributed to vegetable preferences (shared environmental correlation: 0.97), whereas almost three-quarters of these influences were shared between FF and fruit preferences (shared environmental correlation: 0.72). Common shared environmental influences likely include the early family feeding environment, with siblings raised in the same household sharing similar diets, early feeding experiences, and food exposures. Other shared environmental factors such as food availability, common illnesses, and parental modeling may also contribute to the commonalities in children’s food preferences and fussiness.

A considerable proportion of the variance in liking for fruit and vegetables and in FF was also independent insofar as the phenotypic associations were not complete. This unique variance may have reflected distinct mechanisms that are exclusive to each trait, which may also include behavioral or psychological traits such as other appetitive or personality phenotypes. Previous research has shown that individuals who are more sensation seeking tend to be much-less food neophobic (3, 36), possibly because they have lower levels of neophobia in all domains (37). Other personality factors, such as anxiety (3), neuroticism (38) and openness (39), have also been shown to be related to food rejection, and selective eating behaviors have been linked with psychopathologic symptoms including depression and attention-deficit/hyperactivity disorder (40).

To our knowledge, this is the first study to explore the extent to which FF and liking for specific food groups share common genetic influences. The large sample size and multivariate design provided robust estimates for the heritability of FF and also for the shared pathways that influence FF and liking for vegetables and fruit. However, there were several limitations that should be acknowledged. The large sample size prohibited behavioral observations, and parent-reported measures of food liking and FF were necessary because of the young age of the children (41). The children in this study were 3 y old; because FF emerges in early childhood and may peak in the preschool years (42), the findings from this study may reflect a very specific period in development, thereby limiting the wider implications of these results. There is a need for replication of this research at older ages.

In conclusion, this novel investigation into the shared influences that underlie FF and liking for vegetables and fruit in early childhood provides strong evidence that common genetic influences are driving the observed phenotypic associations between these traits. These findings may help to explain why, of all the food groups, vegetables and fruit are the foods that are rejected most often by fussier children.

## Footnotes

7 Abbreviations used: CEBQ, Children’s Eating Behavior Questionnaire; CT/CT, cross-twin, cross-trait; FF, food fussiness; ICC, intraclass correlation; MLSEM, maximum-likelihood structural equation modeling; PROP, 6-*n*-propylthiouracil; *TAS2R38*, taste 2 receptor member 38.
